# Salt Cocrystal of Diclofenac Sodium-L-Proline: Structural, Pseudopolymorphism, and Pharmaceutics Performance Study

**DOI:** 10.3390/pharmaceutics12070690

**Published:** 2020-07-21

**Authors:** Ilma Nugrahani, Rizka A. Kumalasari, Winni N. Auli, Ayano Horikawa, Hidehiro Uekusa

**Affiliations:** 1 School of Pharmacy, Bandung Institute of Technology, Bandung, West Java 40132, Indonesia; kumalsarizka@gmail.com (R.A.K.); winnie.nur.auly@gmail.com (W.N.A.); 2 Department of Chemistry, School of Science, Tokyo Institute of Technology, Tokyo 152-8551, Japan; a.horikawa.chem@gmail.com (A.H.); uekusa@chem.titech.ac.jp (H.U.)

**Keywords:** diclofenac sodium, L-proline, salt cocrystal, multicomponent crystal, monohydrate, tetrahydrate, solubility, dissolution, stability

## Abstract

Previously, we have reported on a zwitterionic cocrystal of diclofenac acid and L-proline. However, the solubility of this multicomponent crystal was still lower than that of diclofenac sodium salt. Therefore, this study aimed to observe whether a multicomponent crystal could be produced from diclofenac sodium hydrate with the same coformer, L-proline, which was expected to improve the pharmaceutics performance. Methods involved screening, solid phase characterization, structure determination, stability, and in vitro pharmaceutical performance tests. First, a phase diagram screen was carried out to identify the molar ratio of the multicomponent crystal formation. Next, the single crystals were prepared by slow evaporation under two conditions, which yielded two forms: one was a rod-shape and the second was a flat-square form. The characterization by infrared spectroscopy, thermal analysis, and diffractometry confirmed the formation of the new phases. Finally, structural determination using single crystal X-ray diffraction analysis solved the new salt cocrystals as a stable diclofenac–sodium–proline–water (1:1:1:4) named NDPT (natrium diclofenac proline tetrahydrate), and unstable diclofenac–sodium–proline–water (1:1:1:1), named NDPM (natrium diclofenac proline monohydrate). The solubility and dissolution rate of these multicomponent crystals were superior to those of diclofenac sodium alone. The experimental results that this salt cocrystal is suitable for further development.

## 1. Introduction

Diclofenac is a well-established non-steroidal anti-inflammatory drug (NSAID) with anti-inflammatory, analgesic, and antipyretic activities [1], and is the most widely consumed NSAID in some countries. In Indonesia, diclofenac is one of the three most widely used NSAID drugs and represents 14.4% of all NSAIDs consumed in the country [2]. Based on the Biopharmaceutics Classification System (BCS), diclofenac is a class II compound with high permeability but low solubility, which results in limited bioavailability. Therefore, it is necessary to increase the solubility of the compound.

Techniques that can improve the solubility and bioavailability of drugs include cosolvation, surfactant usage, pH adjustment, solid dispersion, salt formation, and cocrystallization [3,4,5,6,7]. Pharmaceutical cocrystals are multicomponent systems in which two or more components, in a drug–drug or drug–coformer combination, are present in a stoichiometric ratio and bonded together with hydrogen interactions in a crystal lattice [7]. The rationale behind screening for suitable coformers for pharmaceutical development of a hydrophobic drug should consider the rule of five by Lapinski, i.e., hydrogen bonding, halogen bonding (and non-covalent bonding in general), length of the carbon chain, molecular recognition points, and aqueous solubility [8,9].

Cocrystals can be classified into three groups: anhydrous/non-solvated cocrystal, hydrate/solvate cocrystal, and salt cocrystal [9,10,11]. We are focusing on salt cocrystallization, which is a special kind of cocrystal having a salt structure (anion and cation) with a neutral coformer. It is expected to have a combined benefit of the salt and cocrystal properties, such as improved stability, solubility, dissolution, as well as enhanced the bioavailability [12,13,14,15,16]. As previously reported, salt cocrystal niclosamide and salt cocrystal pefloxacin can enhance solubility and dissolution [17,18]. Moreover, our previous research has shown that a salt cocrystal can be made from saccharine sodium salt with theophylline, as well as acidic saccharine with a similar excipient [19].

Diclofenac acid is a poorly soluble drug. Recently, we have attempted and reported on a cocrystal of neutral diclofenac acid with a zwitterionic coformer, L-proline (LP), which increased the drug solubility by more than 7.6-fold [20]. However, the solubility level was not improved over the salt form, diclofenac sodium, which has been developed and more commonly used in dosage forms at the present time [21,22,23,24,25,26,27,28,29]. Some reports have described that this sodium salt can be found in anhydrous and several hydrated forms, i.e., the tetrahydrate [24,25,26], pentahydrate [27], trihydrate [28], and 4.75-hydrate [29], respectively. These phenomena that cause commercial of anhydrous diclofenac sodium (ND) are often found in the mixture with the hydrate phases (NDH) during distribution and storage [28]. Fortunately, NDH could change back to ND by heating and drying [25,26,28,29].

This study aimed to discover salt cocrystals of NDH with LP (abbreviated NDP) to obtain the drug’s superior properties, namely, stability, solubility, and dissolution [3,4,5,6,7,14,15,16,17,18,19,20]. Considering that ND was unstable, we initially arranged a cocrystal from NDH to develop a suitable and practical method, which can be performed readily even under the ambient conditions. As LP is a zwitterion, it was not expected to form a salt with diclofenac acid moiety. The cationic and anionic groups in this amino acid were predicted to arrange strong hydrogen bonds or charge interactions with other molecules [20]. The most important of LP physicochemical aspects are its high wettability, hydrotropic property, and its flexibility to dissolve in a wide pH range [20,30,31,32]. Besides, LP was selected again in this work due to its safety, economy, and availability.

The experiment began with screening the physical interaction between the compounds using a phase diagram, and then the cocrystal was isolated utilizing a specific molar ratio of components. The cocrystal was characterized by binocular microscope, infrared spectroscopy, thermal analysis, and powder X-ray diffractometry, then it was assessed by single crystal X-ray diffractometry until the final 3D structure was determined. Due to the pseudopolymorphism phenomenon of this new multicomponent cocrystal, the parameters that need to be discussed are the hydrate stability, which was tested by drying and humidifying at specific temperature and humidity levels. The next steps were solubility and dissolution tests. The results of this study are expected to demonstrate that this new phase derived from ND and LP increases the solubility and dissolution of ND and is stable.

## 2. Materials and Methods

### 2.1. Materials

Materials used in this experiment were commercial pharmaceutical-grade of ND (Pharos Indonesia, Semarang, Indonesia); ND-anhydrous pro-analysis 99.9% from Sigma Aldrich (Jakarta, Indonesia); L-proline from Tokyo Chemical Industry (Tokyo, Japan); methanol, ethanol, acetone, and potassium bromide (KBr) crystal for infrared analysis; sodium hydroxide (NaOH); potassium hydrogen phosphate (KH_2_PO_4_); and hydrochloride acid (HCl); these reagents, solvents, and materials were purchased from Sigma Aldrich and Merck (Jakarta, Indonesia). Distilled water was prepared by Bandung Institute of Technology (Bandung, Indonesia) and aluminum pans were obtained from Rigaku (Tokyo, Japan).

### 2.2. Methods

#### 2.2.1. Preparation and Characterization of Starting Materials

The references have stated that ND can easily change to NDH, which was stable in room temperature and atmosphere, causing commercial ND is commonly found in mixture with its hydrates [25,26,28,29]. Therefore, this experiment was started by preparing a homogeneous phase of NDH to develop a method under ambient conditions (condition I) promptly, besides under the restricted low humidity (condition II). NDH was prepared by storing commercial ND (Pharos Indonesia Ltd., Semarang, Indonesia, claimed as anhydrous) in an opened container under ambient temperature (72 ± 2% RH/25 ± 2 °C) for 24 h. Anhydrous ND was purified by storing the commercial ND in a desiccator with silica gel for 24 h for cocrystal preparation under the environment with the minimal water (condition II). NDH, ND, and LP were characterized using a Fourier transform infrared (FTIR) spectrophotometry, differential scanning calorimetry (DSC), differential thermal analysis/thermogravimetry (DTA/TG), and powder X-ray diffractometry (PXRD).

#### 2.2.2. Screening to Determine the Optimal Molar Ratio of Cocrystals Using a Binary Phase Diagram

The physical mixtures of NDH and LP were made in several molar ratios of 10:0, 9:1, 8:2, 7:3, 6:4, 5:5, 4:6, 3:7, 2:8, 1:9, and 0:10, or 0.0–1.0 molar fraction of NDH in the mixture with LP. The ingredients were weighed using a Mettler Toledo microbalance (Greifensee, Switzerland) and mixed gently (to avoid the cocrystal formation in situ) until homogeneous. For each composition, the transition phase temperature was measured using an electrothermal, and the melting point data were plotted against the fraction molar of NDH to make a phase diagram [33,34,35,36].

#### 2.2.3. Preparation of NDP Cocrystals 

The cocrystal preparation was performed using two conditions. First, ~1 g mixture of NDH and LP in 1:1 molar ratio was dissolved in 25 mL of various solvents: ethanol, acetone, and methanol (80–90%), respectively (condition I). The clear solutions were then slowly evaporated under ambient conditions (75 ± 2% RH/25 ± 2 °C). Second, a similar amount of preparation was made from anhydrous ND - LP in a 1:1 molar ratio, which was dissolved in 25 mL volume of the purer ethanol, acetone, and methanol (90–95%) (condition II). The cocrystallization was performed in a fume hood under room temperature.

#### 2.2.4. Electrothermal Measurement

A small amount of sample was filled into a capillary tube and then was put on the sample holder of the Electrothermal AZ 9003 apparatus (Staffordshire, UK). The start temperature on the device was set at 10 °C under the first transition temperature point of each sample, with a heating rate of 10 °C min^−1^. Phase changes, including water releasing, melting point, and decomposition, were observed via the magnification viewer on the apparatus. The temperature related to the sample’s behavior under heating was recorded thoroughly.

#### 2.2.5. Crystal Habit Observation

The habit of the recrystallized NDH, LP, and NDP from Condition I and II were put on the objective glass and were observed under a binocular microscope Olympus CX21, (Tokyo, Japan), without cover glass. The pictures were taken by I-phone 7 camera in magnification 100×.

#### 2.2.6. FTIR Measurement

The amount of 99 mg KBr crystal and 1 mg sample were mixed and compressed to be a disc using a hydraulic presser. The pellet was mounted on the holder, and the infrared spectral measurements were carried out using a FTIR Jasco 4200 Type-A (Easton, PA, USA), over the wavenumber range of 4000 to 400 cm^−1^ at 4 cm^−1^ resolution. The starting materials (NDH and LP), physical mixture 1:1 (PM), and salt cocrystals (NDP) were analyzed. Data were processed by Microsoft Excel 2003 and Origin-Pro 8.5.1 software. The second derivative spectra were generated to enhance the specificity [37,38].

#### 2.2.7. DSC Measurement

DSC measurement was performed using a Rigaku Thermoplus DSC 8230 (Tokyo, Japan) under a nitrogen purge of 50 mL/min. For this, 3–5 mg of sample was placed in a closed aluminum pan and measured in a temperature range of 30 to 350 °C for ND and 30 to 250 °C for LP and the cocrystals, with a heating speed of 10 °C min^−1^. An empty closed aluminum was used as a reference.

#### 2.2.8. DTA/TG Measurement

DTA/TG measurement was performed under a nitrogen purge of 50 mL/min using a Rigaku Thermoplus DTA/TG 8122 apparatus (Tokyo, Japan). For this, 5–10 mg of sample was placed into an open aluminum pan and measured in a temperature range of 30 to 350 °C for NDH and 30 to 250 °C for LP and the cocrystals. An empty open aluminum pan was used as a reference. All measurements were conducted with a heating speed of 10 °C min^−1^.

#### 2.2.9. PXRD Measurement

The sample powder was placed between Mylar^(R)^ film on sample holder of Rigaku SmartLab PXRD (Tokyo, Japan). The powder diffraction pattern was collected from 2θ = 3° to 40° at ambient temperature at the step and scan speeds of 0.01° and 3° min^−1^, respectively, using a Cu-Kα source at 45 kV and 200 mA. The PXRD pattern was plotted using Microsoft Excel 2003 and Origin-Pro 8.5.1 software.

#### 2.2.10. Crystal Structure Analysis Using Single-Crystal X-ray Diffraction Analysis (SCXRD)

Single-crystal X-ray diffraction data were collected in ω-scan mode using a Rigaku R-AXIS RAPID diffractometer (Tokyo, Japan) with a Mo Kα radiation (λ = 0.71075 Å) rotating anode source. The integrated and scaled data were empirically corrected for absorption effects using ABSCOR. The initial structures were solved using direct methods with SHELXT and refined with SHELXL. All nonhydrogen atoms were refined anisotropically. All hydrogen atoms were found in a difference Fourier map; however, they were placed by geometrical calculations and treated using a riding model during the refinement. Two salt cocrystals of diclofenac sodium–proline–water were found and determined successfully, which then were named NDPT (from condition I) and NDPM (from condition II).

#### 2.2.11. Stability Test

NDPT stability was tested under three conditions. The first treatment was drying in a controllable incubator Eyela (Tokyo, Japan), which was set at 30 ± 0.5% RH/40 ± 0.5 °C to observe water release. Sample was taken from the incubator after 0, 2, 4, 6, 12, and 24 h of drying. The second treatment consisted of the NDPT sample at room humidity and temperature (72 ± 2% RH/25 ± 2 °C), which was sampled periodically until 72 h. The third stability test was performed by storing the dried sample at high relative humidity (~94% RH), which was prepared by filling a chamber with a saturated solution of potassium nitrate [16]. Sampling was carried out periodically to investigate the changes of water molecules and hydrate stability for 15 days by DTA/TG, DSC, and PXRD.

#### 2.2.12. Solubility Test

Previously, a calibration curve was composed of a series of concentration of anhydrous ND standard solutions in CO_2_-free distilled water (pH 7.0), which measured by a Beckman Coulter DU720 UV–Vis spectrophotometer (Miami, FL, USA) at a wavelength of 276 nm [1,20,26]. Samples of ND, the PM, NDPM, and NDPT were placed into a 50 mL Erlenmeyer flask, and 10 mL of CO_2_ free distilled water was added. The mixture was stirred on Oregon KJ-201BD shaker (Jiangsu, China) for 8 h at a speed of 100 rpm at 25 ± 2 °C, and the steady saturated equilibrium solubility was maintained. The concentrations of the sample were determined using absorbance conversion through a previously created regression equation.

#### 2.2.13. Dissolution Test

Testing began with making dissolution medium mirroring conditions in the human stomach (pH 1.2) and intestine (pH 6.8). The pH 1.2 medium was prepared from 16.67 mL HCl—12 M, which was added to distilled water (pH 7.0) and was then adjusted to 1L. It was fixed at a pH of 1.2.

The “intestineal” dissolution medium was made from aqueous KH_2_PO_4_ 0.2 M with NaOH, which was dissolved in 250 mL of CO_2_-free distilled water (pH 7.0). This mixture was then added with distilled water until 1 L and adjusted with NaOH and H_3_PO_4_ to a fixed pH of 6.8. The pH values were checked using a pH meter from Mettler Toledo (Jakarta, Indonesia).

A calibration curve for concentration determination was composed previously of a series of standard solutions of ND (anhydrous-pro analysis from Sigma Aldrich) in each medium. The absorbance was measured at λ = 276 nm using the UV–visible spectrophotometer.

Before testing, the diclofenac level of all samples was calculated based on the UV absorbance comparison referred to as the ND standard reference. The equality value was used for weighing the samples, which must be equivalent to 200 mg anhydrous ND. Each powder sample for dissolution testing was sifted using a 120 mesh sieve and then was placed in the basket of Guoming RC-1 dissolution apparatus (Shanghai, China), dipped in the dissolution flask containing 900 mL of medium at a temperature of 37 ± 0.5 °C, and rotated at a speed of 50 rpm. A 2 mL volume of sample was taken after 3, 5, 10, 15, 30, and 45 min. Each sample solution was previously filtered through a 0.45 µm membrane filter before the UV absorbance measurement.

## 3. Results and Discussion

### 3.1. ND and NDH Preparation

The hydrate forms of ND have been extensively reported [24,25,26,27,28,29]. In this experiment, NDH was used as the initial material due to its stability compared to anhydrous ND, as previously stated, to absorbed water from the environment quickly. The instability of anhydrous ND raw material in pharmaceutical manufacturing caused it to mix with the hydrate forms. However, the dehydrated material can be obtained by heating or drying of NDH under specific conditions [25,26,28,29]. Therefore, we considered using NDH as the first starting material for the cocrystal screening instead of ND. First, NDH was prepared by storing the commercial ND (claimed as anhydrous) under ambient conditions (72 ± 2% RH/25 ± 2 °C) for 24 h.

As has been cited in Introduction section, there are some reported hydrate forms. However, FTIR, PXRD, and TG data confirmed that the NDH obtained from this technique was a tetrahydrate, which consisted of 20% water [26,28]. We also checked that even NDH changed to ND by restoring it in the desiccator with silica gel for 24 h (referred to anhydrous ND analysis standard, 99.9%, from Sigma Aldrich, Jakarta, Indonesia), also met the reports [25,26,28]. The NDH has been rechecked to be stable during the experiment, following the works in [25,26,29]. In addition, we also observed that NDH could be dehydrated and back to ND entirely by storing it in a desiccator with silica gel during 24 h. Powder X-ray diffractogram and TG thermogram data of NDH and ND comparing to anhydrous ND analysis standard are attached in Supplementary S1–S3.

### 3.2. Determination of Molar Ratio in NDP for Cocrystallization

The fixed molar ratio of ND and LP for arranging the cocrystal was determined using thermal profile analysis. Modification of covalent bonds in a chemical structure can change its melting behavior. Within the survey, 50 cocrystal samples were analyzed, and 51% of the cocrystals analyzed had melting points between those of the active pharmaceutical ingredient (API) and coformer, while 39% were lower than either the API or coformer. Only 6% had melting points higher than the starting materials, and 4% had melting points equal to either the API or coformer [15]. Thermal analysis through the construction of melting temperature versus the composition on a phase diagram is known to establish whether a combination forms a cocrystal or eutectic [15,33,34,35,36]. A typical binary phase diagram of a eutectic mixture will depict a V shape with one eutectic point. A cocrystal forms a “W” shape, with two eutectic points, and one higher melting point between them.

**Figure 1:**
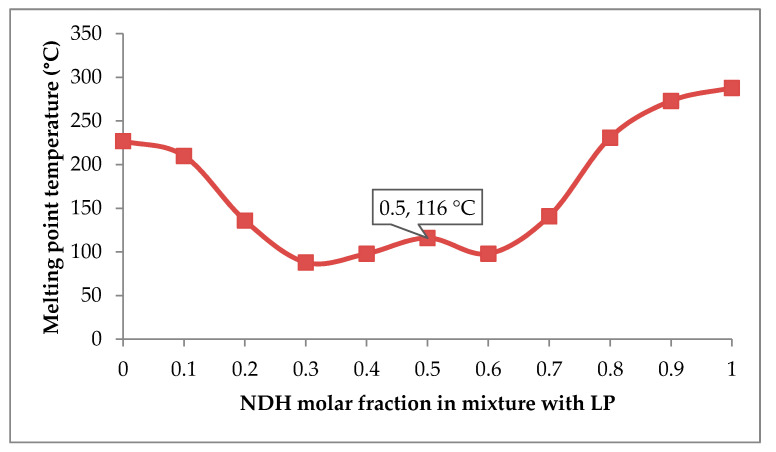
Phase diagram of diclofenac sodium hydrate (NDH) and L-proline (LP) composed from a series of molar ratios, represented by the molar fraction of NDH in the mixture towards the melting point.

The melting temperature data (*y*-axis) of NDH–LP mixtures were plotted against the NDH molar fraction (*x*-axis) to produce a phase diagram (Figure 1). The phase diagram showing a “W” cocrystal typical pattern, with two eutectic points found at the molar fractions of ND 0.3 (at 88 °C) and 0.6 (at 98 °C), respectively. The graphic reveals that the 0.5 molar fraction of ND is the peak, has a higher melting temperature (at 116 °C) than the eutectic points. Therefore, NDH–LP (abbreviated NDP system) could be predicted to form a cocrystal with the 1:1 molar ratio as the fixed stoichiometric proportion.

### 3.3. NDP Cocrystals Preparation and Characterization

#### 3.3.1. Crystal Habit Observation

**Figure 2:**
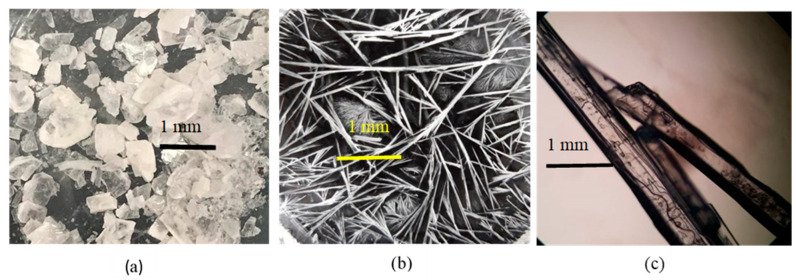
Crystal morphology under a binocular microscope of (**a**) diclofenac sodium hydrate (NDH), (**b**) L-proline (LP), and (**c**) diclofenac–sodium–proline cocrystal (NDP). All recrystallizations were used ethanol solvent 95% under ambient conditions (72 ± 2% RH/25 ± 2 °C).

The crystal morphology was observed as simple identification, which then should be analyzed thoroughly with the more precise solid analysis instruments. Based on the screening data, cocrystals were collected by dissolving the (1:1) molar ratio of NDH–LP mixture in the first condition explained in the Methods section. It was observed that the crystallization of NDP from all solvents occurred fast (less than 24 h) under room temperature and yielded a similar habit and different from the starting materials. As a visualization, Figure 2 shows the crystal picture of the rectangular NDH (a), needle-shaped LP (b), and a cylindrical rods-shaped NDP (c). The different crystal habit of diclofenac–sodium–proline (NDP) from the starting material may indicate the new phase formation, which then should be confirmed thoroughly, by solid characterization using FTIR, PXRD, DSC/DTA/TG, and SCXRD.

#### 3.3.2. FTIR Measurement Data

**Figure 3:**
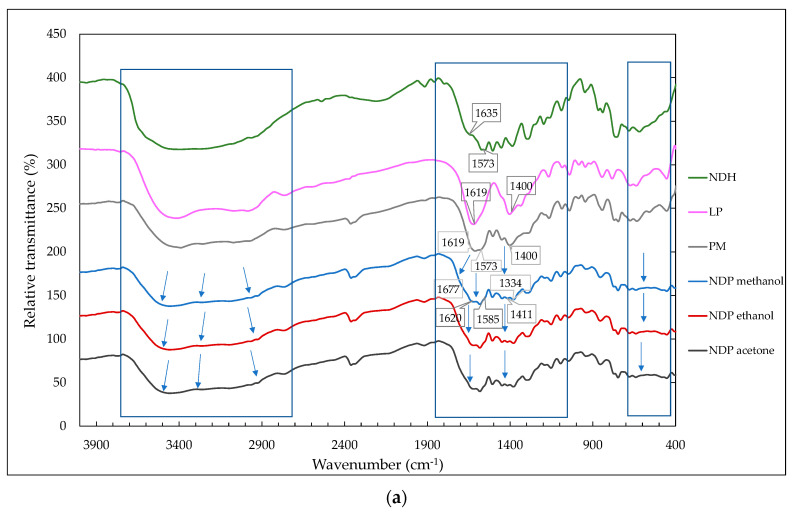

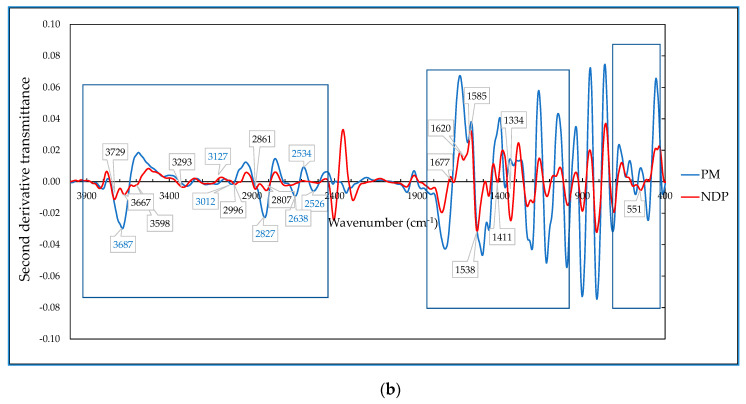
(**a**) FTIR spectra of diclofenac sodium hydrate (NDH), L-proline (LP), physical mixture 1:1 (PM), and the multicomponent 1:1 (NDP) from ethanol, methanol, and acetone. All NDPs have similar spectra, therefore only one of NDP spectra is marked on value of wavenumber changes. (**b**) The 2^nd^ derivative FTIR spectra of NDP and PM. The blue number belongs to PM, and the black number reflects the NDP bands.

In the crystal arrangement, FTIR has commonly used to detect the hydrogen bonding formation [37,38,39,40,41,42,43,44,45]. Figure 3a reveals infrared spectra of NDH, LP, physical mixture 1:1 (PM), and NDP crystals from ethanol, methanol, and acetone, respectively. FTIR spectra of NDH indicated the water –OH stretching band at 3200–3500 cm^−1^ and COO^−^ stretching band at 1631 and 1573 cm^−1^ [21,26]. LP depicts the C=O stretching at 1619 cm^−1^ and COO^−^ stretching of amino band at 1400 cm^−1^ [20,37]. Next, similar spectra were shown by NDP cocrystals obtained from all solvents (methanol, ethanol, and acetone). It can be seen that the cocrystal spectra were different from PM in several bands between 4000 and 400 cm^−1^ regions of measurement.

Due to some overlapped bands, the second derivative spectra (Figure 3b) was generated to increase specificity by separating the overlapped bands on the similar region [37,38]. There are three focused areas shown in Figure 3, with the distinctive bands assigned by the wavenumber point and arrow.

Figure 3b shows the broadband in the region of NDP at 2800–3700 cm^−1^, which was known as the position of –OH stretching from water and carboxylic groups [26,39]. The -OH water molecule bands of NDH have been previously reported to exist on 3200–3500 cm^−1^, coordinated with Na^+^, and interacted by the hydrogen bond to the other molecules [26]. The functional group’s broadband at 3200–3750 cm^−1^ region also may involve –NH and NH–O stretching. Meanwhile, 1600-1750 cm^−1^ included –NH bend and –C=O stretching of carboxylic from both starting materials of the intermolecular bond [21,26,38,39,40,41,42,43,44,45,46].

In more detail, derivative spectra of PM and cocrystal in Figure 3b show that the new bands at 3598, 3667, and 3729 cm^−1^ of NDP replaced the 3687 cm^−1^ band of PM, which indicated the change in OH stretching. Next, here were new NDP bands at 2807 and 2861 cm^−1^ and the disappearing of 2827, 2526, and 2638 cm^−1^ bands from PM. These data revealed the change of –OH stretching of carboxylic of the two materials due to the cocrystal formation. In addition, the distinctive bands at 1620 cm^−1^ and 1677 cm^−1^ on the NDP spectra also support the new bonding formation.

The other changes revealed were the shifting of the C=O band at 1573 cm^−1^ to 1585 cm^−1^ and the amino stretching band from 1400 cm^−1^ to 1411 cm^−1^. This data reflected the alteration of COO^−^ vibration of NDH and amino sidechains of LP [21,37,38]. A sharp band on 1334 cm^−1^ also supported the data of new interaction that involved –OH and C–O carboxylic. Besides, the changes of the 500–600 cm^−1^ band confirmed that –NH and CO moiety of LP contributed to composing the cocrystal. Therefore, overall, FTIR data supported a new solid structure construction, which was arranged by the carboxylate and amine from both starting materials, Na^+^ ions from ND, and water molecules.

#### 3.3.3. Thermal Analysis

Next, the electrothermal measurement on NDH showed a water spot at 70–100 °C, decomposed from 239.5 °C (change to a yellow mass), and finally melted at 288 °C. LP melted at 227 °C, as well as NDH, was continued by decomposition. Meanwhile, the new crystals released globules of water from about 50 °C, became very wet at ~86 °C, melted at ~116 °C as a clear liquid, and decomposed after ~200 °C, indicated by the color change and gas release. Thus, it can be concluded that the new cocrystal had a lower melting point than the starting components. In addition, all samples decomposed after melting.

**Figure 4:**
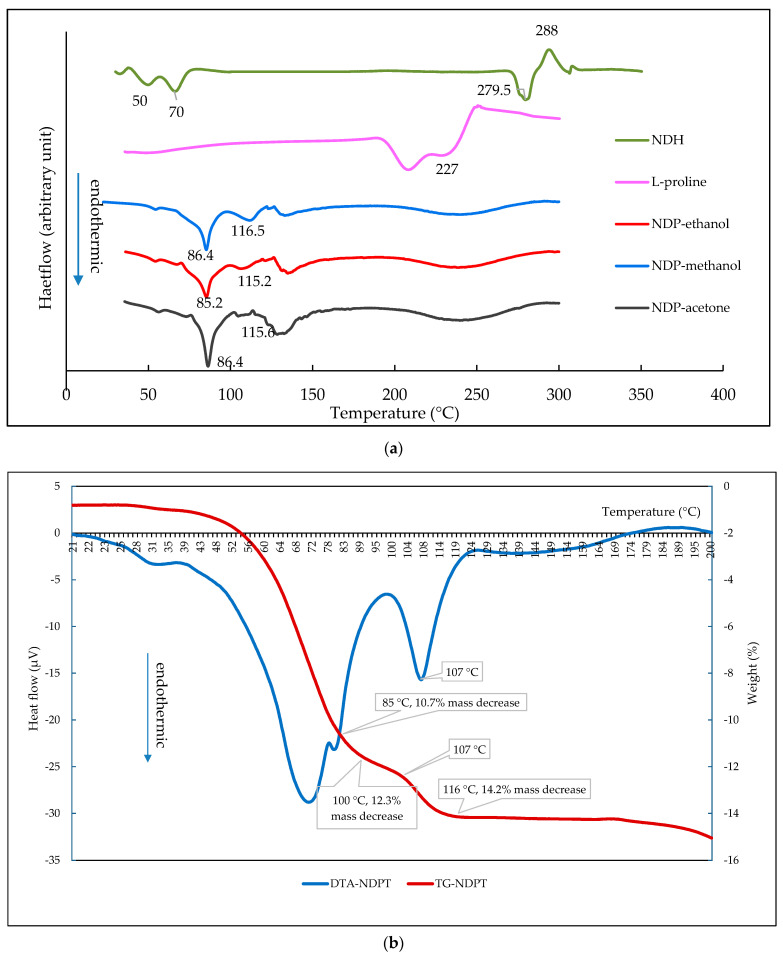
(**a**) Differential scanning calorimetry (DSC) thermogram of diclofenac sodium hydrate (NDH); L-proline (LP); and NDP cocrystals from acetone, ethanol, and methanol, respectively. (**b**) DTA and TG thermogram of NDP, shown ~14.2% mass decrease or equal to 4 water molecules.

The DSC and DTA/TG measurement results are presented in Figure 4. Figure 4a depicts a DSC thermogram overlay of NDH, LP, and NDP crystals. Figure 4a shows that NDH had endothermic curves at 50 and 70 °C, which indicates water release, next it melted at ~280 and oxidized at 288 °C. This data met the reference of ND tetrahydrate, as well as the FTIR spectra [26]. Meanwhile, LP melted and decomposed at 227 °C. Next, all thermograms of NDP cocrystals from aqueous 90% of methanol, ethanol, and acetone in this figure reveal the similar first endothermic peaks at 86.4, 85.2, and 85.3 °C; and the second peaks at 114.5, 115.2, and 116.5 °C, respectively. The first curves are predicted as the first step of water release, and the second curves indicate the last part of the water molecule release, which concurrently occurs with the melting point. It can be seen here that the melting point of NDP at 114–116 °C was significantly lower than the starting components (288 and 227 °C). The samples decomposed after 200 °C, which was indicated by an endothermic curve of DSC/DTA thermogram and the sharp loss of mass, as shown by the TG thermogram. These data in line electrothermal analysis results. The decrease of melting point reflects hydrogen bond formation between the compounds with the water molecule, which contributed crucially. The new thermal profile can be expected to impact on other physicochemical properties such as solubility, dissolution rate, and stability [14,15,20,47,48,49,50,51].

The DSC thermogram was then elaborated with DTA/TG data in Figure 4b, which shows that the mass decrease of NDP cocrystal was ± 14.2% *w/w* or equal to four water molecules. Hereafter, this new phase was estimated as a tetrahydrate form, named diclofenac sodium proline tetrahydrate (NDPT). A small difference in the transition temperature was shown between DSC and DTA/TG. The water release and melting point temperature on DTA/TG thermograms are shown similar to electrothermal data, but the endothermic peak of DTA was depicted lower than DSC. This difference was caused by the open pan used in DTA/TG versus the closed pan used in the DSC measurement.

The open pan used in DTA/TG measurement facilitated the loss of water freely, as did the open capillary tube used in the electrothermal study. Therefore, the endothermic peak of the melting point on the DSC thermogram was at the end of the DTA endothermic peak and the transition point of TG thermograms, 116 °C. Following the specification and the standard operation of the procedure analysis, the closed pan was preferred to maintain the DSC’s instrument durability. DSC is more sensitive than DTA and highly influenced by liquid or gas contaminants. Prior analysis using electrothermal, which was elaborated with DTA/TG, showed that ND, LP, and the NDP cocrystal broke down and released water and gas; therefore, the closed pan was the best choice for DSC analysis.

The relationship between hydrate release and cocrystal melting mechanism of NDP can be studied from the DTA (red) and TG (blue) thermogram. The TG thermogram in Figure 4b shows that 10.7% mass, equal to three water molecules, decreased sharply in the temperature range of 50 to 85 °C. This following by a flatter curve until 100 °C, which shows the weight decreased up to 12.3% *w/w*, or equal to 3.5 water molecules. Finally, the last step of loss water, which demonstrated on the thermogram as −2% of weight, equivalent to half moles of water, was evident at 100–116 °C. This final water release was related to the endothermic peak at 107 °C on the DTA thermogram, which concurrently indicated the melting point of the cocrystal. These thermal data led to the prediction that the water molecules interacted in the different strengths in NDPT, and the last part of the water release was responsible for the cocrystal breaking.

#### 3.3.4. PXRD Data

**Figure 5:**
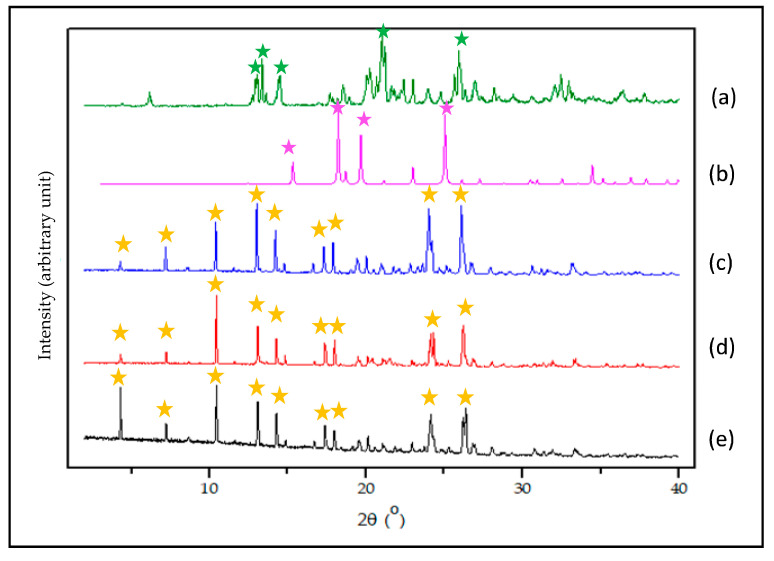
Diffractogram of NDH (**a**); LP (**b**); and NDPT from the 95% of methanol (**c**), acetone (**d**), and ethanol (**e**). The distinctive peaks of the new phase are assigned with yellow mark.

Figure 5 depicts the diffractograms of NDH, LP, and NDPT cocrystals from three kinds of solvent. NDH is shown in Figure 5a to have the specific peaks at 2θ = 13.1°, 13.8°, 15.0°, 22.2°, and 26.5° (assigned by green marks). It was supported by the previous FTIR and TG data confirmed as a tetrahydrate, with water mass ~20% [26]. Next, it is shown in Figure 5b that the LP diffractogram had specific peaks at 2θ = 15.2°, 18.0°, 19.5°, 24.8° (purple marks). The diffractograms NDPT from all solvents that are shown in Figure 5c–e were similar, with the distinctive peaks are demonstrated at 2θ = 4.3°, 7.2°, 10.4°, 13.0°, 14.2°, 17.9°, 20.1°, 24.1°, and 26.3° (yellow marked). These data confirmed that a new multicomponent phase was produced from NDH and LP.

### 3.4. NDPT Crystal Structure Determination

**Figure 6:**
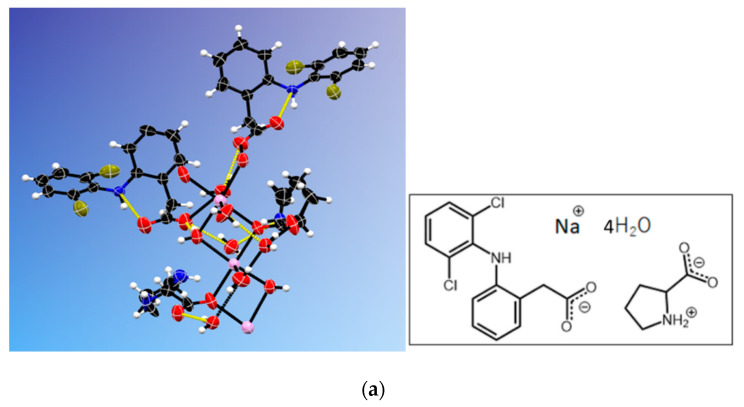

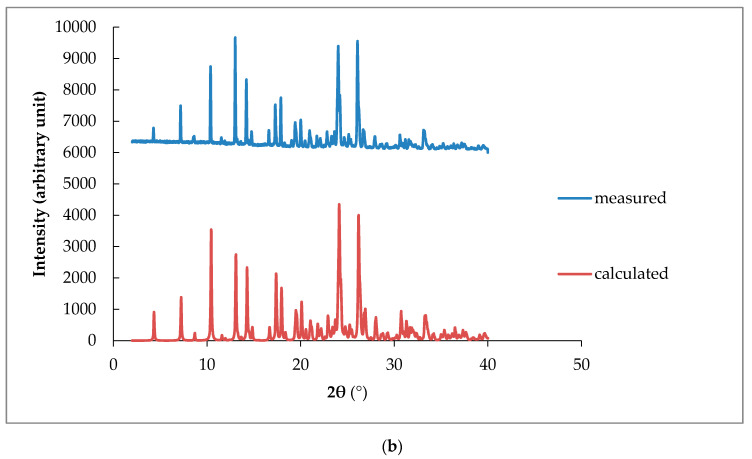
(**a**) ORTEP cell view and molecular structure graph of NDPT; Na^+^ is in violet. (**b**) The measured and calculated diffractograms of NDPT.

The crystal structure of NDPT was determined by single-crystal X-ray analysis entirely, resulting in crystallographic data, which are listed in Table 1. From the study, NDP multicomponent crystal was confirmed to contain equimolar amounts of ND and LP. Furthermore, this crystal was found to be a hydrate with four water molecules for one molecule of ND and LP. Thus, it met with the TG result and was fixed to be a tetrahydrate. The salt cocrystal structure is depicted in Figure 6a, with the diffractogram is shown in Figure 6b.

The crystallographic data of NDPT are listed in Table 1, which revealed that this new phase is a monoclinic crystal with the space group *P*2*_1_*.

**Figure 7:**
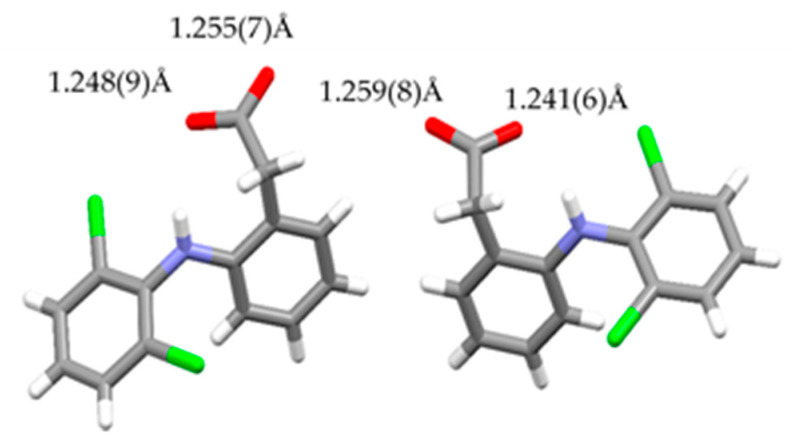
C–O distances in diclofenac molecules in NDPT.

Considering the atomic distance in the NDPT crystal structure, as shown in Figure 7, the C–O distance indicated that the diclofenac molecule was ionized. Based on this analysis, it can be concluded that the new crystal phase was a salt cocrystal, arranged from an ionized drug (diclofenac sodium) with a neutral coformer (LP) and supported by the water molecules as the bridge between the components. This interaction was different from the previous cocrystal of neutral diclofenac acid with LP [20]. The alkaline and ionized diclofenac can be expected to increase solubility and dissolution more than the neutral cocrystal, which then was proven further.

**Figure 8:**
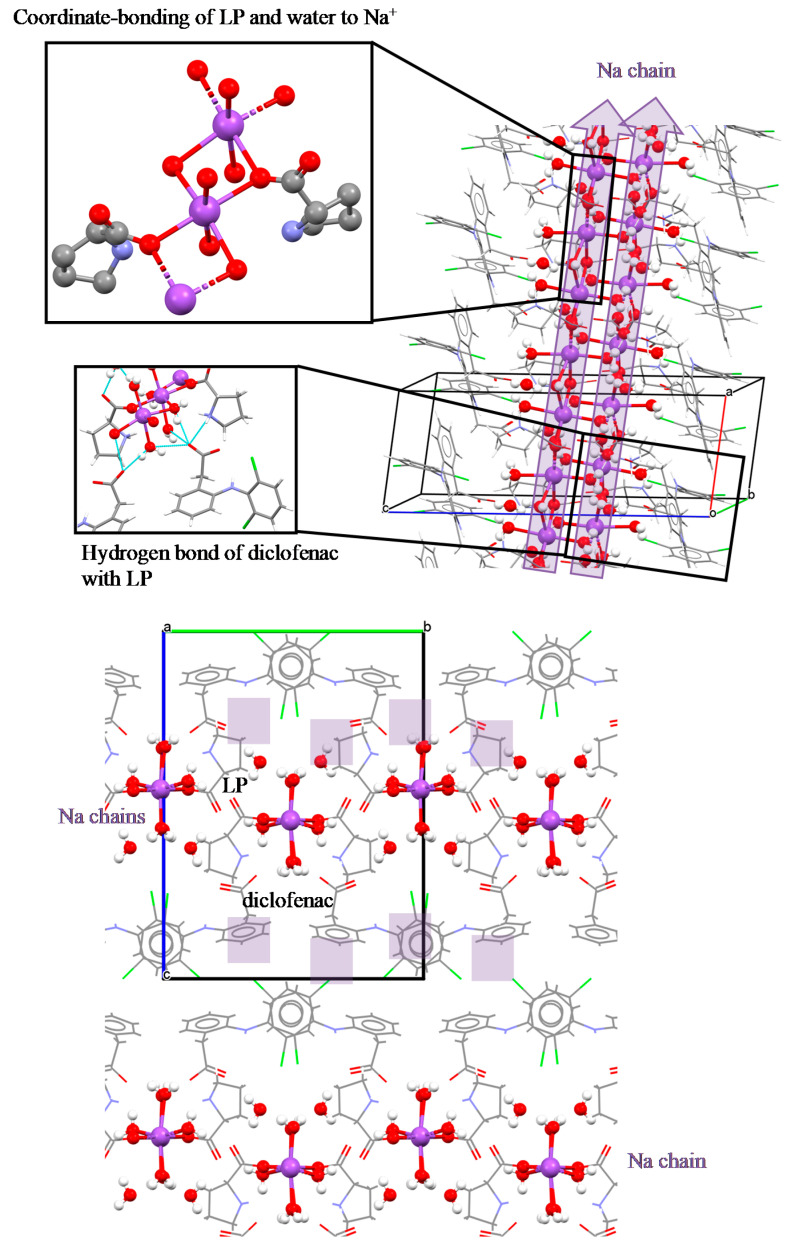
The Na chain structure in NDPT (diclofenac–sodium–proline–tetrahydrate), Na^+^ is in violet.

Figure 8 presents the unique conformation of each component in the tetrahydrate salt cocrystals, which involves sodium, diclofenac, proline, and water. In the asymmetric unit of this crystal, there are two ND and two LP molecules and eight water molecules. From these water molecules, six molecules coordinate to Na^+^, and two molecules form hydrogen bonds with other water molecules, LP molecules, and diclofenac molecules without coordination to Na^+^. In addition, two LP molecules were also coordinated to Na^+^, which was a 6-coordinated structure totally. These coordination bonds formed a one-dimensional (along a-axis) chain consisting of Na atom and water molecules, and both diclofenac molecules were connected to this Na chain by hydrogen bonds with water molecules. Furthermore, the carboxy oxygen of the diclofenac molecule formed a hydrogen bond with the nitrogen atom of LP. The formation of this hydrogen bond (C=O ··· HN) was also detected by an FTIR band at 1620 cm^−1^ and the change of 500–600 cm^−1^ band. The LP molecule was disordered at the 5-membered ring.

### 3.5. Diclofenac Sodium-Proline Monohydrate Salt Cocrystal Isolation, Characterization, and Structure Determination

**Figure 9:**
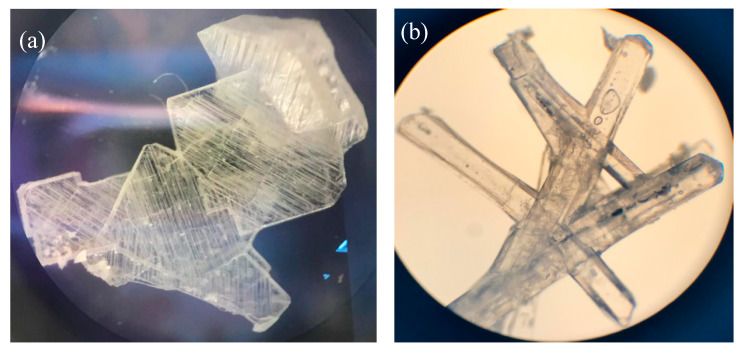
Crystal of the new diclofenac–sodium–proline–monohydrate (**a**) compared to NDPT (**b**) under a binocular microscope observation (100×).

We attempted to discover the other hydrate form of NDP by arranging the different circumstances of cocrystal production, called condition II. This condition was performed by storing all starting materials in a desiccator with silica gel for 24 h (0% RH/25 ± 2 °C) to minimize water existence. FTIR, TG, and PXRD were used to evaluate the solid state of ND and LP. As a result, we observed that even NDH back to ND, in line with the works in [25,26]. Based on the previous data of NDPT, the water molecule was shown to be prominent in the intermolecular interaction. From a screening, the starting materials of ND and LP were still found when the pure solvent (≥98%) was used. Thus, next cocrystal was collected using the purer solvents than that was used to produce NDPT, but it should still provide a small amount of water, i.e., the aqueous 90–95% *v/v* organic solvents. From this condition, a new crystal habit differed to NDPT was obtained successfully. The photographs present the newest crystalline morphology in Figure 9 (left) compared to the previous structure, NDPT, in Figure 9 (right).

**Figure 10:**
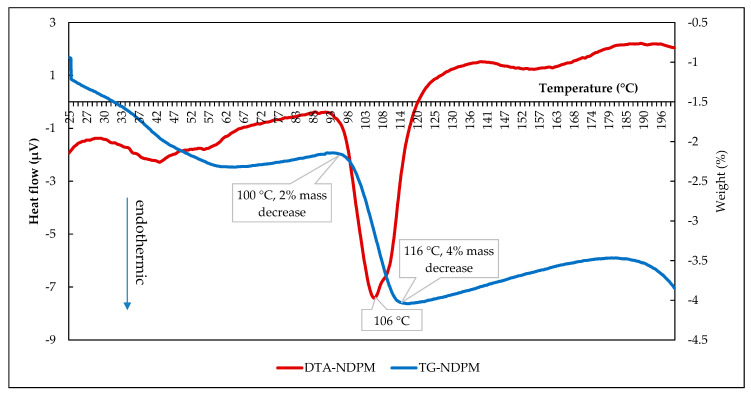
DTA and TG thermograms of NDPM.

The DTA/TG results of the newest cocrystal are revealed in Figure 10 below. Red and blue curves represent DTA and TG thermograms, respectively, which show the water release from room temperature to 200 °C. Approximately 2% of water released gradually at 30–100 °C, followed by a rapid loss of the left of ~ 2% water molecule (equal to 0.5 moles of water) at 100–116 °C, which occurred together with the melting point shown by a peak on DTA thermogram at 106 °C. It means that NDPM’s melting point was not significantly different from NDPT. The total water release was ~4% *w/w*, or equal to a monohydrate. Therefore, it was named diclofenac sodium–proline–monohydrate (NDPM). Based on TG data, as well as shown in the NDPT thermogram, half moles of water molecule interacted strongly with the other components in NDP, which released lastly by breaking down the cocrystal completely.

**Figure 11:**
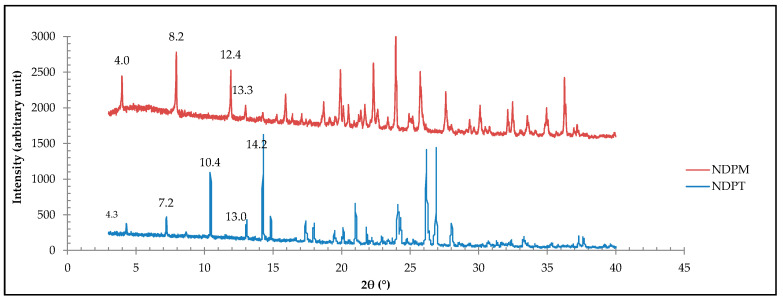
The measured diffraction pattern of NDPM (red) shows the different profile compared to NDPT (blue).

PXRD analysis collected the diffractogram pattern of NDPM, which is depicted in Figure 11, showing the distinguished peaks at 2θ = 4.0°, 8.2°, 12.4°, and 13.3°; compared to NDPT peaks at 2θ = 4.3°, 7.2°, 10.4°, and 13.0°. This data confirmed that the new crystal obtained from the condition II was a different phase than NDPT, which then was determined further by SCXRD.

**Figure 12:**
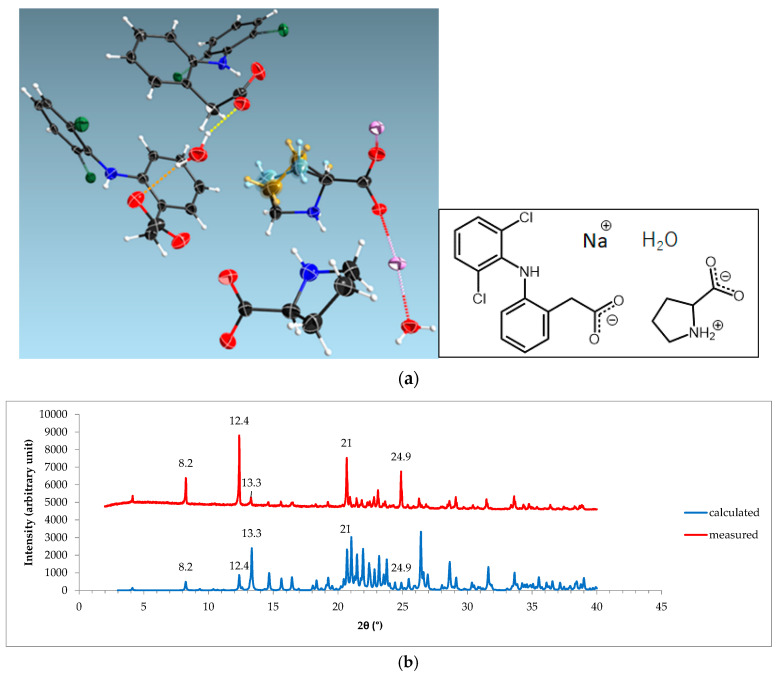
(**a**) ORTEP cell view and molecular structure graph of NDPM; Na^+^ is in violet. (**b**) The measured and calculated diffractograms of NDPM.

The structure determination using SCXRD at −180 °C confirmed that the new phase was a monohydrate, which consisted of diclofenac–sodium–proline–water (1:1:1:1). The ORTEP draw of NDPM structure is depicted in Figure 12a. In addition, Figure 12b reveals the similarity of the empirical and calculated diffractogram. Thus, the data show that NDPM structure has been determined entirely.

**Figure 13:**
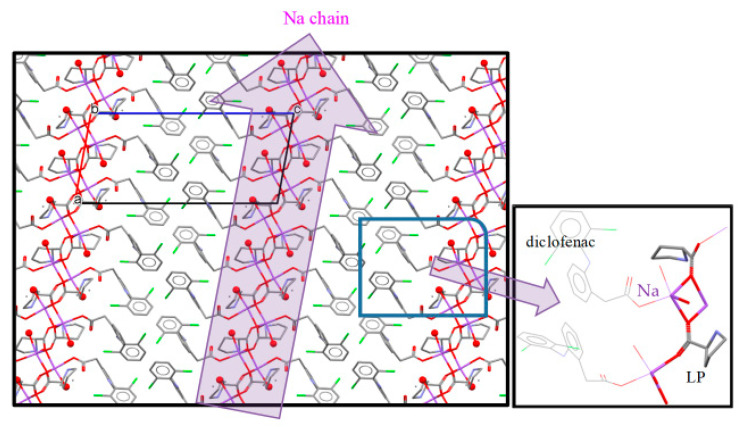
The order of diclofenac–sodium–proline (NDP) system. L-proline (LP) allows Na^+^ to form a coordination work with diclofenac site. Na^+^ is in violet.

The detailed element configuration of NDPM is shown in Figure 13. This figure presents that diclofenac is coordinated around the Na^+^ network to form a layer structure. Meanwhile, LP allowed Na^+^ to build a coordination network. However, the data of NDPM were not as perfect as the NDPT data, due to the instability and very thin crystal habit of this phase. Crystal NDPM was shown to be unstable and immediately transformed into NDPT, indicated by the habit change of the cocrystal under ambient conditions. This property was confirmed by TG/DTA and PXRD, which will be discussed further in Section 3.6 (Stability Testing).

The crystal data of NDPM is listed in Table 2 below.

In conclusion, the multicomponent of ND with LP was found in two forms, which were tetrahydrate (NDPT) and monohydrate (NDPM) salt cocrystals. The crystal data of NDPT and NDPM have been submitted to Cambridge Crystallographic Data Center (CCDC) with the deposit number 1938590 and 1983478, respectively. At the present time, the anhydrous phase is under investigation and not yet developed because it is difficult to compose, even in its powder form. This fact underlies the prediction that the presence of water is crucial in the formation of hydrogen bonds in this new salt cocrystal multicomponent system.

### 3.6. Stability Testing

Several stability studies are typically performed during the development of cocrystals, including drying and humidifying studies. The type of stability test depends on the structural characteristics of the sample [20,23,52,53,54]. Due to the four water molecules and the two LP molecules coordinated to Na^+^ (sodium), it can be predicted that NDP can be transformed from one hydrate to the other phase easily, as well as ND [23,24,25,26,27,28,29]. The existence of a cationic element, mainly alkaline, has been well known to absorb water molecules quickly and release the water under dry conditions. Therefore, the hydrate stability test under drying and extreme humidity were essential to perform.

#### 3.6.1. Thermal Profile Analysis on Dried Stability Test Samples

**Figure 14:**
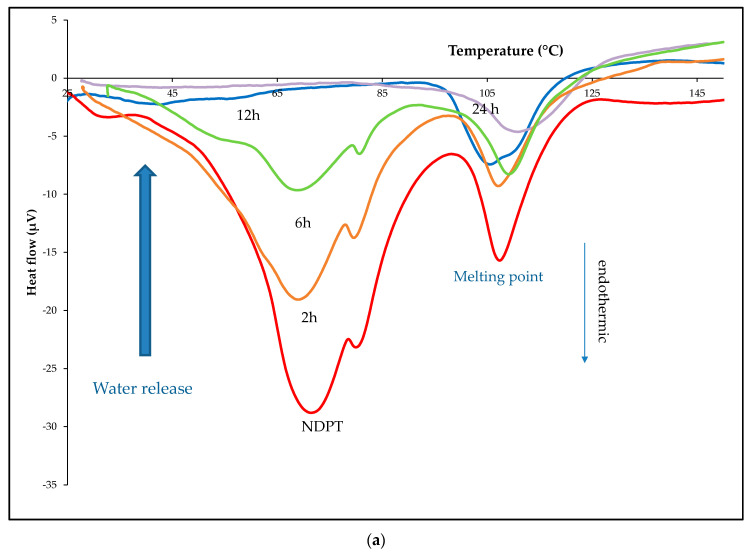

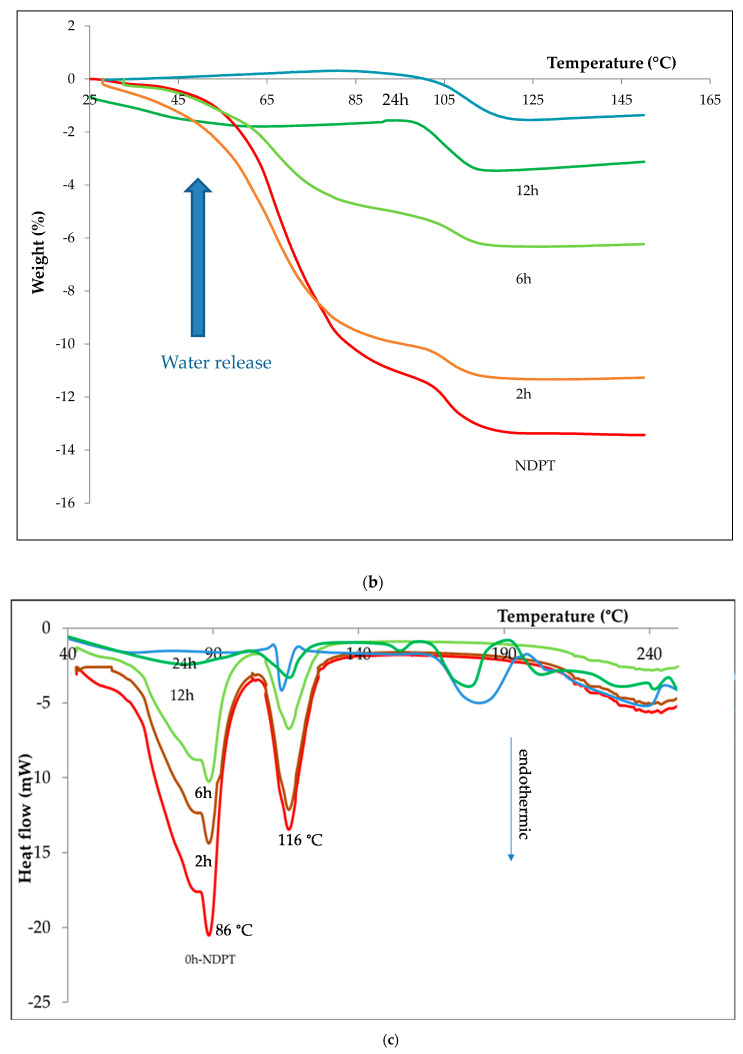
DTA (**a**), TG (**b**), and DSC (**c**) thermograms of NDPT sample that was stored in the incubator 30% RH/40 °C for 0, 2, 6, 12, and 24 h.

Dried samples were evaluated using DTA/TG/DSC and the data are shown in Figure 14. Figure 14a is DTA thermogram of the samples, show the decreasing of dehydrated curve at ~30–100 and 100–125 °C by the heating. Next, the TG thermograms in Figure 14b show the release of water of the hydrate at 2–6 h, leaving approximately one water molecule after 12 h or equal to a monohydrate salt cocrystal. It finally lost almost all water molecules after 24 h of drying in a controllable incubator, set at 30% RH/40 °C. DSC analysis was also conducted to confirm the thermal data of the new phases. The obtained thermograms are presented in Figure 14c, which are equal with the DTA/TG profile. These data were then elaborated with PXRD analysis, which will be discussed in Section 3.6.3.

#### 3.6.2. Thermal Profile Analysis on Humidified Stability Test Samples

**Figure 15:**
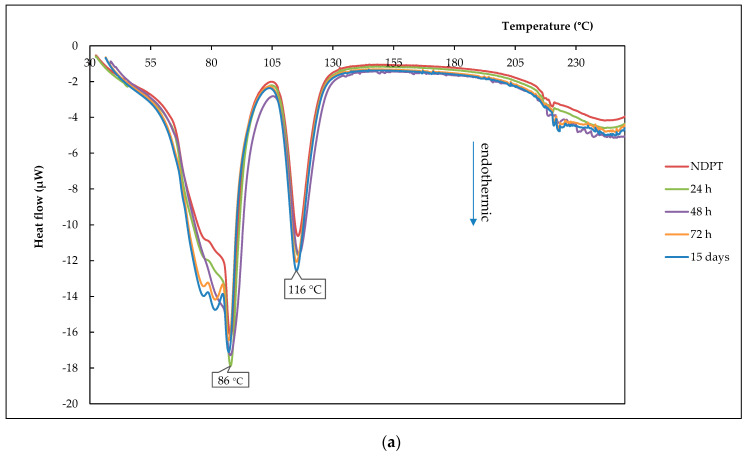

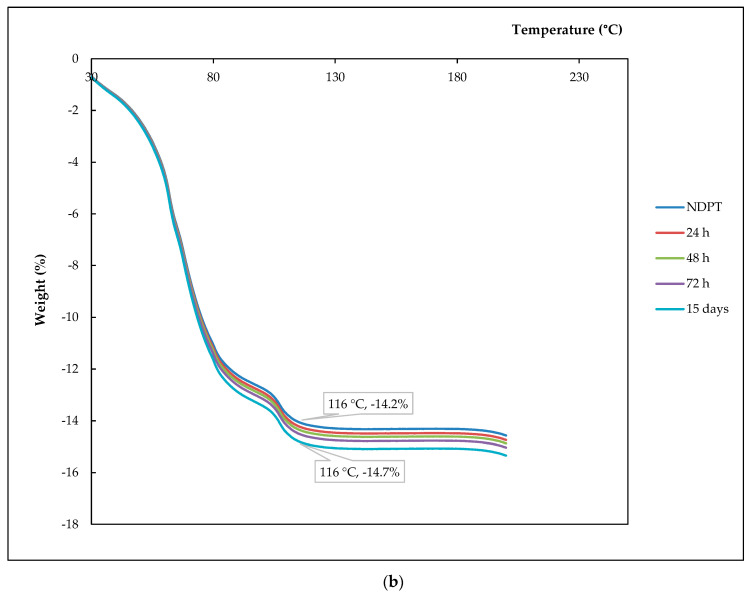
DSC (**a**) and TG (**b**) thermograms of NDPT after storage at high relative humidity of 94% RH/25 ± 2 °C for 24, 48, 72 h, and 15 days.

Figure 15a shows the NDPT cocrystal thermograms from DSC after 24, 48, and 72 h of storage in a chamber of 94 ± 2% RH/25 ± 2 °C. The first endothermic peak at ~86 °C is a water release point and the second endothermic peak is shown at ± 116 °C. The slightly different profile of the 72 h and 15 days samples were shown in the first endothermic curve, separated into two small peaks. However, TG thermograms in Figure 15b reveal that the water portion did not increase significantly after 15 days, which was ~0.5% *w/w* or equal to 0.1 moles of water molecules only. This data indicated that the tetrahydrate was still stable. PXRD analysis confirmed NDPT stability under this humid condition, as explained in Section 3.6.3, concurrently with the drying stability test result.

#### 3.6.3. PXRD Analysis of Stability Test Samples after Drying and Humidifying

**Figure 16:**
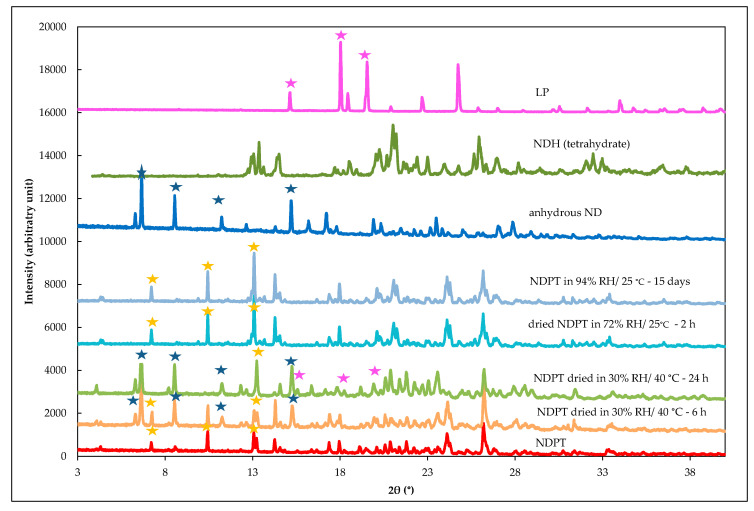
Diffractogram of NDPT fresh preparation after drying in 30% RH/40 °C for 6 h and 24 h, dried NDPT after 2 h restored in 72 ± 2% RH/25 ± 2 °C, and dried NDPT after restored in a 94% RH/25 ± 2 °C chamber for 15 days. Anhydrous ND, NDH (tetrahydrate), and LP diffractograms were depicted as the comparison. Assigned peaks represent ND (blue ticks), LP (purple ticks), and NDPT (yellow ticks).

PXRD was then used to confirm the thermal analysis data from the stability test on NDPT samples, which were collected both by drying and storage in humid conditions, shown in Figure 16. ND, NDH, and LP diffractograms were depicted as the reference. Previously, it was predicted that the drying of NDPT would produce NDPM, as did by heating NDH to produce ND [25,26]; however, the diffractogram data revealed that the dried sample after 6 h of storage at 30 ± 0.5% RH/40 ± 0.5 °C showed a mixture peaks of NDPT and the starting materials, i.e., ND and LP, without any less hydrate of NDP. In this figure, the specific peaks of samples are assigned by the ticks in yellow (NDPT), blue (NDH), and green (LP). Moreover, NDPT changed to a similar diffraction pattern with a mixture of the components, anhydrous ND and LP, which are depicted in the figure as the comparison, after 12–24 h of drying. The ND peaks at 2θ = 6.7°, 8.6°, 15.2°, and 17.2° (blue ticks) appeared in the dried NDPT samples, as well as LP peaks at 2θ = 15.2°, 18.0°, 19.5°, and 24.8° (purple ticks). The final mixture did not contain NDH due to the dry condition desorbed all water molecules. These data describe that water loss broke cocrystal binding and did not result in another new phase, including NDPM. Thus, the water molecule was crucial in the NDPT system to mediate the interaction between components in this tetrahydrate salt cocrystal.

Inversely, Figure 16 also shows that after 2 h restored under ambient conditions (72 ± 2% RH/25 ± 2 °C), the diffractogram of the sample was back to the NDPT profile, with the specific peaks at 2θ = 4.3°, 7.2°, 13.0° (yellow ticks), without producing NDPM. Moreover, NDPT was still stable under high humidity, i.e., 94 ± 2% RH/25 ± 2 °C, until 15 days, indicated by the steady diffractograms. In conclusion, NDPT was a stable phase, comparable to NDH pseudo polymorphs’ stability, which has been reported to be more stable than ND [25,26,28,29]. Anhydrous ND changed rapidly under ambient conditions to its hydrate (NDH) [25,26], similar to NDPM, which quickly transformed into NDPT. However, it differs from NDH-ND transformation, which can occur easily by drying under the dry condition (30% RH/40 °C), NDPT could not transform to the less hydrate by this manner.

### 3.7. Pharmaceutics Performance Tests

The pharmaceutical performance of the new salt cocrystals was carried out by investigating the impact of the multicomponent formation on ND solubility and the dissolution profile. The tests were performed with NDPM and NDPT, compared to anhydrous ND.

#### 3.7.1. Solubility Testing

Solubility is of utmost importance in the pharmaceutical field because the drug must dissolve before being absorbed. Table 3 depicts the solubility data for ND, NDPM, and NDPT. The solubility of the physical mixture of anhydrous ND with LP was conducted to represent the dried system, which was not found as the monohydrate cocrystal. Based on the solubility test results, after the equilibrium state, diclofenac was dissolved more readily from NDPM and NDPT, i.e., 4.08 and 3.31-fold increase compared ND solubility, respectively. NDPM was revealed to have higher solubility than NDPT, comparable to anhydrous ND behavior, which was superior to ND hydrate due to the lower of crystal lattice energy with a wider space between molecules [24,25,26,27,28,29]. The increased solubility of the NDP systems was related to the decrease in lattice energy, which promoted the breaking down of solid intermolecular bonds immediately [20,46,48,49,50,51,54].

In addition, the characteristic crystal structure associated with solubility in the NDP salt cocrystals was in the presence of a region consisting only of Na^+^ ions and water molecules (Na chain) that has a sufficiently higher affinity for water than diclofenac acid and LP. Because of this higher affinity, Na^+^ ions and water molecules would be dissolved first through the chain region. As a result, the entire NDP crystal breaks up and dissolves (reduction in lattice energy stabilization). Therefore, the presence of the Na chain and the LP layer would be one of the reasons why the solubility was higher than that of ND. Several studies have reported that such a layer or channel structure consisting only of higher solubility molecules improves the solubility of API multicomponent crystals [50,51].

#### 3.7.2. Dissolution Testing

**Figure 17:**
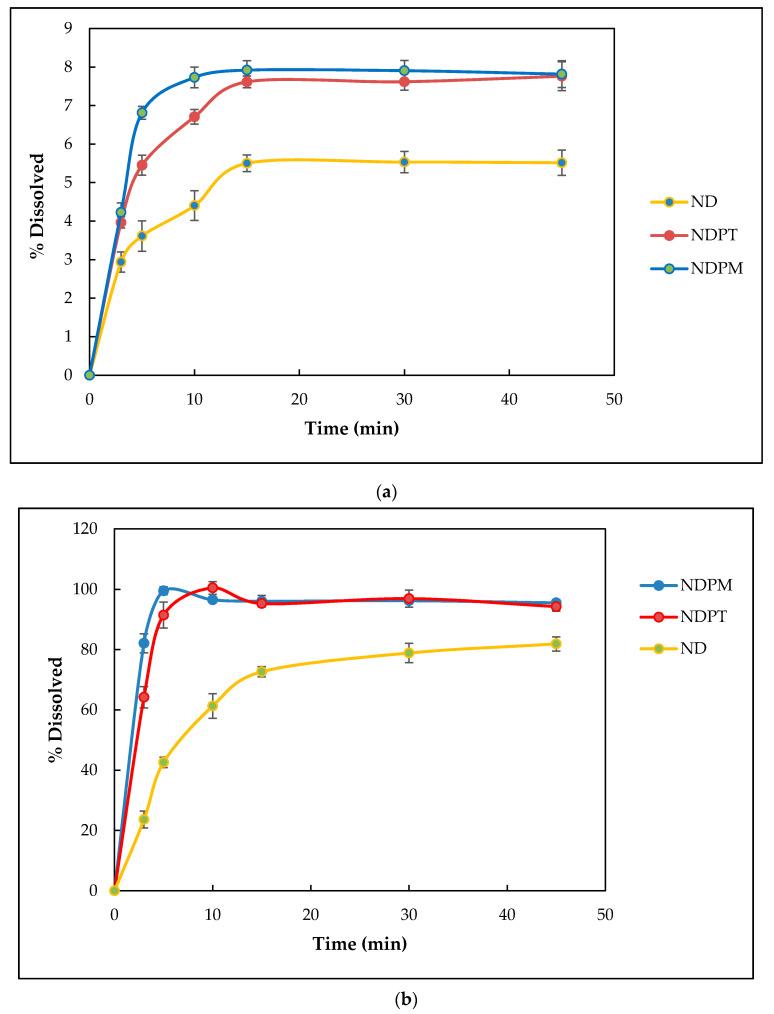
Dissolution profile of anhydrous diclofenac sodium (ND), salt cocrystal diclofenac–sodium–proline–monohydrate (NDPM), and salt cocrystal diclofenac–sodium–proline–tetrahydrate (NDPT) in (**a**) gastric pH (1.2) medium and (**b**) intestinal pH (6.8) medium.

Dissolution testing was performed to observe how salt cocrystal formation affected the ND release rate. Tests were carried out with samples equivalent to 200 mg ND in 900 mL gastric medium (pH 1.2) and intestinal medium (pH 6.8) [25,26,48,55,56,57]. Diclofenac solubility has been reported to be influenced by pH medium and has the optimum dissolution in pH neutral to base [25,48,57]. Based on dissolution profiles on pH 1.2 medium (Figure 17a), the maximum drug release from all samples (ND, NDPM, and NDPT) was achieved after 15 min dissolution, but in a very low concentration. This result was in line with the information from references [26,49,58]. Due to the acidity of ND (pKa: 4, at 25 °C), it showed that in the gastric medium, each sample was not completely separated because hydrogen ions inhibited diclofenac dissociation. However, NDPM was superior in increasing the percentage of drug release. Based on the Henderson–Hasselbach’s equation at a pH below the pKa of ND, the percentage of non-ionized substance will be greater than at a pH above the pKa so that the percentage of substances absorbed into the solute tends to increase [55,57,58].

ND dissolved in the pH 6.8 medium. However, it needed time, which was represented by the dissolution profile in Figure 17b. This figure shows that NDPM dissolution was fastest at intestinal pH, which reached a maximum concentration (100% released of 200 mg drug) after 5 min. NDPT needed only 10 min. Meanwhile, the ND curve still increased after 45 min. This means that NDPM was superior in releasing diclofenac in pH 6.8 medium compared to the single ND and the NDPT. As shown in this figure, the existence of LP can push the dissolution, even in the physical mixture. However, the cocrystal formation made the interaction between ND and LP intensively by a stronger hydrogen bonding than the physical mixture. The very immediate entire release of NDPT occurs because the hydroxyl ion can accelerate the dissociation of diclofenac.

The increase in diclofenac solubility from NDPM and NDPT was due to the ability of LP to arrange the coordinate binding with Na^+^ and interact as a zwitterion molecule with diclofenac acid. This extraordinary double action of LP facilitated contact between the diclofenac moiety and the dissolution medium, in addition to the dissociation of Na^+^ with diclofenac molecule in the media. By increasing dissolution, NDP salt cocrystals can be expected to improve diclofenac bioavailability [6,59]. The dissolution profile of the salt cocrystals was better than the previous non-salt cocrystal of diclofenac–proline [20], as the sink dissolution conditions can lead to problems with the absorption step of the drug [47,60] did not exist. ND had a good dissolution profile in the buffered pH 6.8 medium, but the cocrystals were superior. The amphoteric ability of LP may adjust the pH to the optimum environment for diclofenac dissolution (the medium pH was measured as 6.8–7.0 after the final dissolution), along with hydrotropic property, which contributes to improve dissolution. NDPM showed a faster dissolution than NDPT for a similar reason to the solubility data. The monohydrate crystal lattice is smaller and has a looser and more spacious space compared to the tetrahydrate form, as presented in Figure 8 and Figure 14, making it dissolve faster. This data was in accordance with anhydrous ND, which has a higher solubility than its hydrate forms [26,28,29].

## 4. Conclusions

ND and LP form two pseudo-polymorphs of multicomponent salt cocrystals. The first multicomponent crystal involves four water molecules, or a tetrahydrate, named NDPT. Meanwhile, the second consists of one water molecule, or a monohydrate, named NDPM. Both hydrates have a monoclinic lattice system. However, the monohydrate form was unstable and changed to NDPT easily under ambient condition. NDPT was a stable salt cocrystal even after breaking the structure after drying due to water loss; its structure recovered rapidly under ambient conditions. Moreover, NDPT structure was also stable under high humidity. Both salt cocrystals significantly increased the solubility and dissolution rate of the original starting material, ND, but NDPM was superior. Further research should hold continuously to observe other polymorphs and hydrates, which is still very challenging. The investigation of diffusion and absorption should also be carried out.

## Figures and Tables

**Table 1:** Crystallographic data of NDPT.

**Crystal name**	Diclofenac-Sodium-Proline-Tetrahydrate (NDPT)
**Moiety formula**	C_19_H_27_Cl_2_N_2_NaO_8_, (C_14_H_10_Cl_2_NO_2_Na) (C_5_H_9_NO_2_) 4(H_2_O)
**Crystal system**	Monoclinic
**Space group**	*P*2*_1_*
**a (+)**	7.6073(3)
**b (Å)**	15.2652(7)
**c (Å)**	20.5263(9)
**Β (°)**	97.602(1)
**V (Å3)**	2362.71(18)
**Z/Z’**	4/2
**T (K)**	296
**R-factor (%)**	5.93

**Table 2:** Crystallographic data of NDPM.

**Page:** 17 **Crystal Name**	Diclofenac-Sodium-Proline-Monohydrate (NDPM)
**Crystal System**	Monoclinic
**Moiety formula**	C_19_H_21_Cl_2_N_2_NaO_5_, (C_14_H_10_Cl_2_NO_2_Na) (C_5_H_9_NO_2_) (H_2_O)
**Space group**	*P*2*_1_*
**a/Å**	9.8240(3)
**b/Å**	9.2835(3)
**c/Å**	21.7931(6)
**β/°**	100.383(2)
**V/Å^3^**	1955.01(10)
**Z/Z’**	4/2
**R/%**	7.43

**Table 3:** Solubility curve (in CO_2_-free distilled water, pH 7.0) of ND, NDPM, and NDPT.

Sample	Solubility (mg/mL) *n* = 3	SD	Solubility Magnitude of ND	pH of Solution
ND	16.18	0.46	1.00	6.9–7.0
NDPM	66.09	1.65	4.08	7.0–7.1
NDPT	53.61	0.83	3.31	7.0–7.1

## Supplementary Materials

The following are available online at https://www.mdpi.com/1999-4923/12/7/690/s1: S1: CIF crystal data of NDP monohydrate, S2: CIF crystal data of NDP tetrahydrate, S3: Diffractogram (1) and TG-thermogram (2) of ND and NDH.

## Abbreviations

ND	natrium diclofenac (=diclofenac sodium)
NDH	natrium diclofenac hydrate (=diclofenac sodium hydrate)
LP	L-proline
NDP	natrium diclofenac proline
NDPT	natrium diclofenac proline tetrahydrate
NDPM	natrium diclofenac proline monohydrate
DSC	differential scanning calorimetry
DTA	differential thermal analysis
TG	thermogravimetry
FTIR	Fourier transform infrared
PXRD	powder X-ray diffractometer/powder X-ray diffractometry
SCXRD	single crystal X-ray diffractometry/single crystal X-ray diffractometer
RH	relative humidity
