# Supplementary material for: Salt Cocrystal of Diclofenac Sodium-L-Proline: Structural, Pseudopolymorphism, and Pharmaceutics Performance Study
Source: Pharmaceutics. 2020 Jul 21;12(7):690. doi: 10.3390/pharmaceutics12070690 (PMC7408265; doi:10.3390/pharmaceutics12070690)

checkCIF/PLATON report

Structure factors have been supplied for datablock(s) shelx_a

THIS REPORT IS FOR GUIDANCE ONLY. IF USED AS PART OF A REVIEW PROCEDURE FOR PUBLICATION, IT SHOULD NOT REPLACE THE EXPERTISE OF AN EXPERIENCED CRYSTALLOGRAPHIC REFEREE.

No syntax errors found. CIF dictionary Interpreting this report

Datablock: shelx_a

```
Bond precision:  C-C = 0.0089 A          Wavelength=0.71075

Cell:            a=7.6073(3)     b=15.2652(7)       c=20.5263(9)
                 alpha=90        beta=97.602(1)     gamma=90
Temperature:     296 K
```

	Calculated	Reported
Volume	2362.71(18)	2362.71(18)
Space group	P 21	P 21
Hall group	P 2yb	P 2yb
Moiety formula	C10 H30 N2 Na2 O10, 2(C14 H10 Cl2 N O2), 2(H2 O)	C38 H38 Cl4 N4 Na2 O8 , 8(H2O)
Sum formula	C38 H54 Cl4 N4 Na2 O16	C38 H54 Cl4 N4 Na2 O16
Mr	1010.63	1010.63
Dx,g cm-3	1.421	1.421
Z	2	2
Mu (mm-1)	0.340	0.340
F000	1056.0	1056.0
F000’	1057.82	
h,k,lmax	9,19,26	9,19,26
Nref	10769[5588]	10695
Tmin,Tmax	0.916,0.960	0.762,0.960
Tmin’	0.915	

```
Correction method= # Reported T Limits: Tmin=0.762 Tmax=0.960
AbsCorr = MULTI-SCAN

Data completeness= 1.91/0.99      Theta(max)= 27.438

R(reflections)= 0.0593( 7586)     wR2(reflections)= 0.1686( 10695)

S = 1.109                  Npar= 733
```

The following ALERTS were generated. Each ALERT has the format
test-name_ALERT_alert-type_alert-level.
Click on the hyperlinks for more details of the test.

Alert level C

```
PLAT094_ALERT_2_C Ratio of Maximum / Minimum Residual Density ....       2.20 Report
PLAT242_ALERT_2_C Low    'MainMol' Ueq as Compared to Neighbors of        Na1 Check
PLAT242_ALERT_2_C Low    'MainMol' Ueq as Compared to Neighbors of        Na2 Check
PLAT242_ALERT_2_C Low    'MainMol' Ueq as Compared to Neighbors of        C14 Check
PLAT242_ALERT_2_C Low    'MainMol' Ueq as Compared to Neighbors of        C28 Check
PLAT340_ALERT_3_C Low Bond Precision on  C-C Bonds ...............    0.00886 Ang.
PLAT910_ALERT_3_C Missing # of FCF Reflection(s) Below Theta(Min).          8 Note
PLAT975_ALERT_2_C Check Calcd Resid. Dens.  0.63A   From O13             0.67 eA-3
PLAT975_ALERT_2_C Check Calcd Resid. Dens.  0.54A   From O13             0.56 eA-3
PLAT975_ALERT_2_C Check Calcd Resid. Dens.  0.98A   From O10             0.50 eA-3
PLAT975_ALERT_2_C Check Calcd Resid. Dens.  0.87A   From N1              0.45 eA-3
```

Alert level G

```
PLAT002_ALERT_2_G Number of Distance or Angle Restraints on AtSite         51 Note
PLAT003_ALERT_2_G Number of Uiso or Uij Restrained non-H Atoms ...         17 Report
PLAT004_ALERT_5_G Polymeric Structure Found with Maximum Dimension          1 Info
PLAT007_ALERT_5_G Number of Unrefined Donor-H Atoms ..............         10 Report
PLAT042_ALERT_1_G Calc. and Reported MoietyFormula Strings  Differ     Please Check
PLAT111_ALERT_2_G ADDSYM Detects New (Pseudo) Centre of Symmetry .         94 %Fit
PLAT112_ALERT_2_G ADDSYM Detects New (Pseudo) Symm. Elem         a         94 %Fit
PLAT113_ALERT_2_G ADDSYM Suggests Possible Pseudo/New  Space Group      P21/c Check
PLAT172_ALERT_4_G The CIF-Embedded .res File Contains DFIX Records         17 Report
PLAT178_ALERT_4_G The CIF-Embedded .res File Contains SIMU Records          1 Report
PLAT186_ALERT_4_G The CIF-Embedded .res File Contains ISOR Records          3 Report
PLAT301_ALERT_3_G Main Residue  Disorder ..............(Resd  1  )        46% Note
PLAT720_ALERT_4_G Number of Unusual/Non-Standard Labels ..........         26 Note
PLAT860_ALERT_3_G Number of Least-Squares Restraints .............        154 Note
PLAT883_ALERT_1_G No Info/Value for _atom_sites_solution_primary .     Please Do !
PLAT912_ALERT_4_G Missing # of FCF Reflections Above STh/L=  0.600         27 Note
PLAT978_ALERT_2_G Number C-C Bonds with Positive Residual Density.          2 Info
```

 0 **ALERT level A** = Most likely a serious problem - resolve or explain
 0 **ALERT level B** = A potentially serious problem, consider carefully
11 **ALERT level C** = Check. Ensure it is not caused by an omission or oversight
17 **ALERT level G** = General information/check it is not something unexpected

 2 ALERT type 1 CIF construction/syntax error, inconsistent or missing data
15 ALERT type 2 Indicator that the structure model may be wrong or deficient
 4 ALERT type 3 Indicator that the structure quality may be low
 5 ALERT type 4 Improvement, methodology, query or suggestion
 2 ALERT type 5 Informative message, check

It is advisable to attempt to resolve as many as possible of the alerts in all categories. Often the minor alerts point to easily fixed oversights, errors and omissions in your CIF or refinement strategy, so attention to these fine details can be worthwhile. In order to resolve some of the more serious problems it may be necessary to carry out additional measurements or structure refinements. However, the purpose of your study may justify the reported deviations and the more serious of these should normally be commented upon in the discussion or experimental section of a paper or in the "special_details" fields of the CIF. checkCIF was carefully designed to identify outliers and unusual parameters, but every test has its limitations and alerts that are not important in a particular case may appear. Conversely, the absence of alerts does not guarantee there are no aspects of the results needing attention. It is up to the individual to critically assess their own results and, if necessary, seek expert advice.

Publication of your CIF in IUCr journals

A basic structural check has been run on your CIF. These basic checks will be run on all CIFs submitted for publication in IUCr journals (*Acta Crystallographica*, *Journal of Applied Crystallography*, *Journal of Synchrotron Radiation*); however, if you intend to submit to *Acta Crystallographica Section C* or *E* or *IUCrData*, you should make sure that full publication checks are run on the final version of your CIF prior to submission.

Publication of your CIF in other journals

Please refer to the *Notes for Authors* of the relevant journal for any special instructions relating to CIF submission.

PLATON version of 17/03/2019; check.def file version of 04/03/2019

Datablock shelx_a - ellipsoid plot

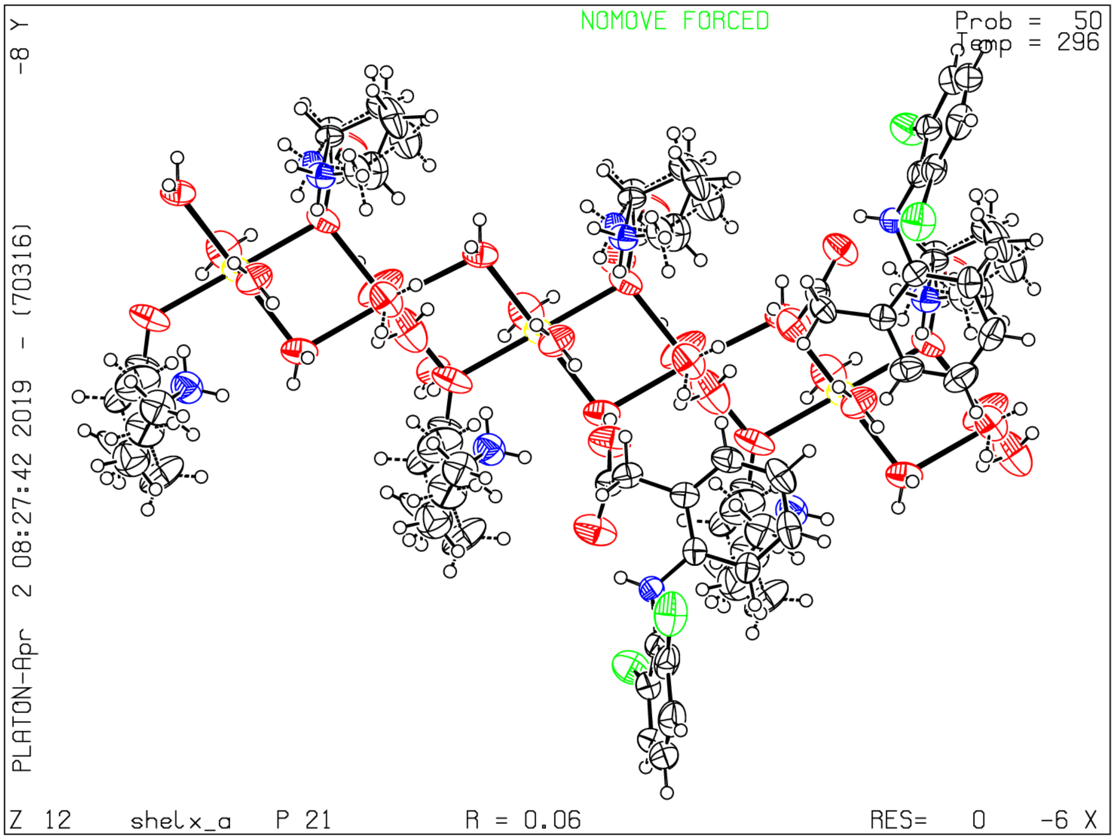

Supplement: Supplementary file 1 [file pharmaceutics-12-00690-s001.zip › checkcif NDP tetrahydrate.pdf]
