# Supplementary material for: Salt Cocrystal of Diclofenac Sodium-L-Proline: Structural, Pseudopolymorphism, and Pharmaceutics Performance Study
Source: Pharmaceutics. 2020 Jul 21;12(7):690. doi: 10.3390/pharmaceutics12070690 (PMC7408265; doi:10.3390/pharmaceutics12070690)
Supplement: Supplementary file 1 [file pharmaceutics-12-00690-s001.zip › Supplementary 3 - PXRD and TG ND and NDH.docx]

Supplementary 3

S3(1): Diffractogram of ND and NDH

S3(2): TG-thermogram of ND and NDH
